# Palatal Obturator after Maxillectomy following Squamous Cell Carcinoma

**DOI:** 10.1155/2022/5545333

**Published:** 2022-01-18

**Authors:** Karim Chebbi, Khaoula Bouaziz, Oumaima Tayari, Azza Berkaoui, Mohamed Ali Bouzidi, Jamila Jaouadi

**Affiliations:** Complete Removable Prosthodontic Department, Research Laboratory LR12ES11, Faculty of Dental Medicine, University of Monastir, Monastir, Tunisia

## Abstract

After surgical excision of tumors involving the maxilla, depending on their location and size, maxillary defects can have harmful consequences, both esthetic and functional. These effects disrupt all the functions of the manducatory system, namely breathing, swallowing, and especially phonation, thus affecting negatively the patient's psychological state. Despite the evolution of reconstructive surgical techniques and the development of microsurgery, conventional obturator prostheses are still relevant. In fact, these prostheses restore the main functions of chewing, phonation, and swallowing. They also provide the patient with a satisfactory esthetic appearance. Moreover, they have an advantage in regard to oncology, making the possibility of surveying much easier. Maxillary defects are characterized by their highly polymorphic aspect, having a great impact on the nature of prosthetic rehabilitation. The aim of this work was to present the different clinical and laboratory steps of prosthetic rehabilitation of an acquired maxillary defect following excision of a mucoepidermoid carcinoma.

## 1. Introduction

Mucoepidermoid carcinoma is a common salivary tumor with varying potential for aggressive behavior [1].

In malignant tumors, mucoepidermoid carcinoma remains the most frequently observed tumor in the salivary glands. The average age of discovery varies between 40 and 60 years depending on the authors. The occurrence in young children and adolescents remains rare, around thirty cases have been described in the literature since 1952 [2].

The classification of mucoepidermoid carcinoma as high-grade connotes the potential for disease progression and possibly disease-related mortality. In addition to complete resection, postoperative adjuvant radiotherapy is indicated for high-grade mucoepidermoid carcinoma [1].

The grading of this tumor has evolved over time from descriptive two-tiered schemata to more objective three-tiered systems. Patients with mucoepidermoid carcinoma classified as high-grade (grade 3) are at significant risk for presenting with positive lymph nodes and developing disease progression, and possibly disease-related mortality [1].

The morphological and spatial characteristics of the tumor, its extension to the neighboring structures, the presence of metastases, the involvement of the lymph node, and the patient's general condition determine the therapeutic choices [3].

The management of mucoepidermoid carcinoma depends on the pathological diagnosis. Surgical treatment remains the therapy of choice. It consists of excision of the tumor with a mucosal and bone carcinological margin. Lymph node dissection is indicated in high-grade tumors where the risk of lymph node metastases is greater than 50% [2].

Its treatment includes surgery associated or not with radiotherapy depending on the histological grade [2].

Surgery can cause maxillary defects, eventually leading to oronasal or orosinusal communication. It must be followed by a prosthetic rehabilitation that allows the patient to regain his manducatory functions [4].

In this work, through a case report, the specificity of prosthetic rehabilitation of a patient with an acquired maxillary defect requiring the placement of an obturator prosthesis was presented.

## 2. Observation

A 65-year-old man, smoker, hypertensive, non-insulin–dependent diabetic, and with a history of hyperthyroidism was referred by the Cancerology department of Salah Azaiez hospital in Tunis (Tunisia) to the department of Maxillofacial Prosthodontics at the Dental Clinic of Monastir (Tunisia) for prosthetic rehabilitation.

Resection of a high-grade squamous cell carcinoma of the palate was performed two years earlier. The patient was also subjected to underwent adjuvant chemotherapy and radiotherapy on the tumor zone and the lymph node areas.

The chief complaint was functional: chewing problems, nasal leakage of fluids, and improper speech.

Extraoral examination revealed a facial symmetry, an absence of cervical lymphadenopathy, a moderately sufficient mouth opening, and a straight mouth opening/closing path.

Intraoral examination revealed the presence of the remaining natural teeth (11, 12, 13, 21, 22, and 23) in the maxilla. These teeth were vital and showed generalized physiological attrition.

The osteomucosal-bearing surface showed a moderate deep palate and a high and wide ridge on the right side, covered with a thick, adherent fibromucosa.

**Figure 1:**
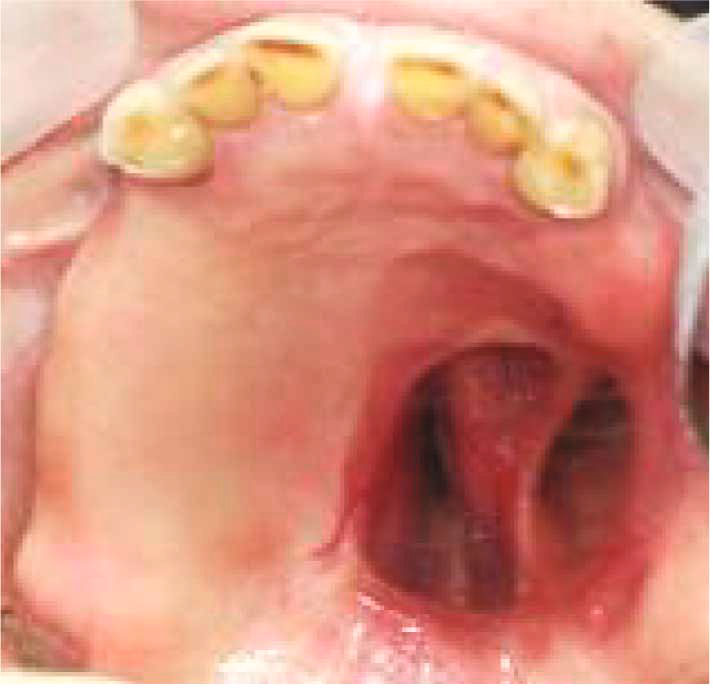
Maxillary defect was connecting the oral cavity to the left maxillary sinus and the nasal fossae.

On the left side, an existing surgical maxillary defect with adequate healing was noticed in the hard palate. This defect was connecting the oral cavity to the left maxillary sinus and the nasal fossae (Figure 1). It also presented two moderately deep anterior and posterior undercuts.

**Figure 2:**
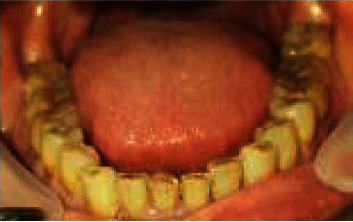
Mandibular teeth showed generalized physiological attrition.

In the mandible, all the teeth were vital and showed generalized physiological attrition (Figure 2).

**Figure 3:**
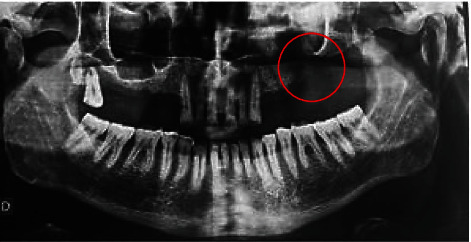
Panoramic radiography. The maxillary defect is shown with the circle above.

**Figure 4:**
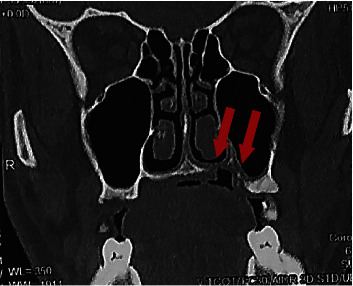
Cone-beam computed tomography (coronal cut) showing the maxillary defect, connecting the oral cavity to the left maxillary sinus and the nasal fossae.

The radiological examination panoramic radiography (Figure 3) and cone-beam computed tomography (CBCT) (Figure 4) confirm the existence of the maxillary defect.

Examination of occlusion showed an unpreserved Occlusal Vertical Dimension (OVD) and Maximum Intercuspid Occlusion (MIO).

The treatment plan involved an obturator prosthesis.

**Figure 5:**
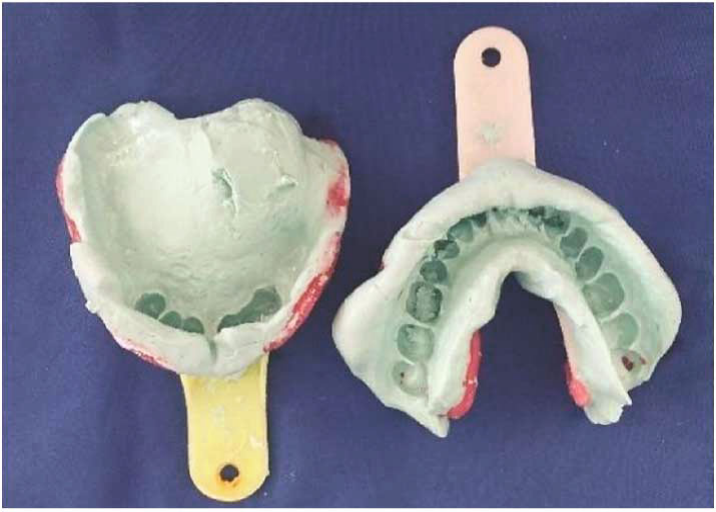
Primary impressions.

Mucostatic primary impressions were made with irreversible hydrocolloid (Alginate) using perforated stock trays, whose sizes were carefully determined (Figure 5). Before taking the maxillary impression, the anterior and posterior undercuts of the maxillary defect were filled with a gauze.

Some adjustments were made using wax, which has been added to the peripheral outline of the upper stock tray on the right side in order to record the vestibular depth as well as the right paratuberosity pocket.

The maxillary impression was poured with white plaster and a custom tray was made with light-curing resin, served as a support for the anatomo-functional impression.

For the mandibular impression, the cast was made with yellow plaster, and it was used for articulator mounting.

The maxillary custom tray was carefully adjusted in the mouth. After validation of the peripheral seal using thermoplastic paste (Kerr®) next to the edentulous ridges, perforations facing the palatal defect were made.

**Figure 6:**
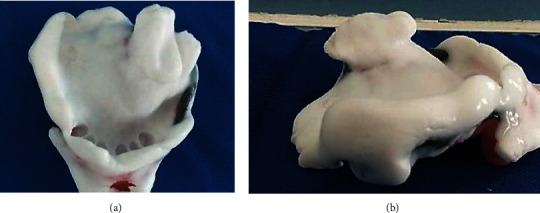
Anatomofunctional impression with FITT material: (a) upper view; (b) lateral view.

**Figure 7:**
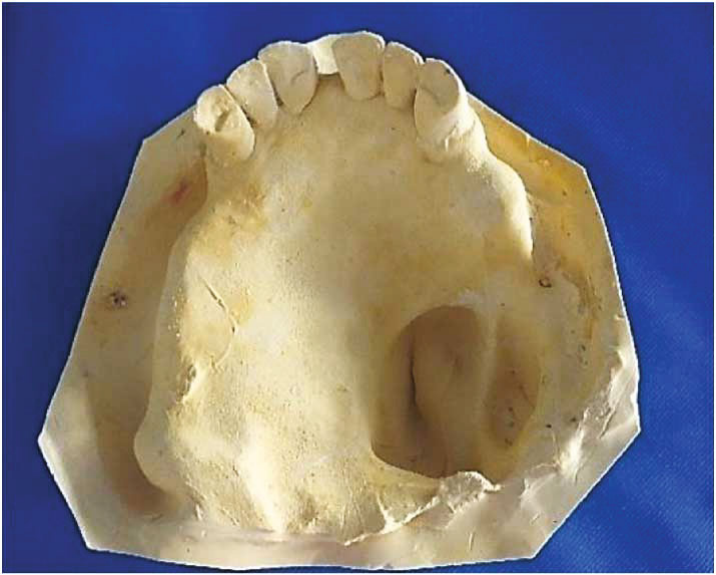
Maxillary cast.

The upper anatomofunctional impression was taken with FITT material (Functional Impression Tissue Toner) (Kerr®) (Figures 6(a) and 6(b)) and was poured with yellow plaster (Figure 7).

Jaw relation was made in centric relation and in the correct occlusal vertical dimension. It was followed by mounting the casts on the articulator and the selection of the artificial teeth color.

**Figure 8:**
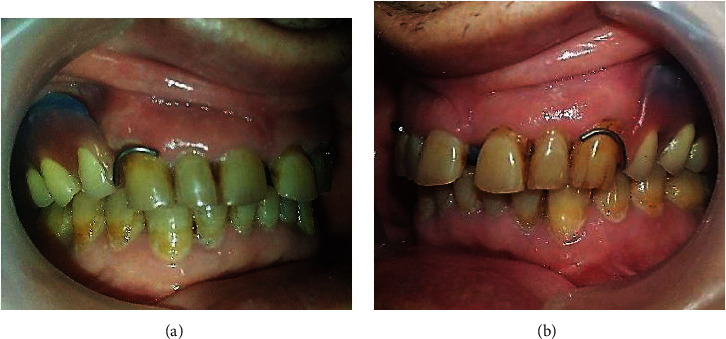
Try-in. (a) Right side. (b) Left side.

After setting up the teeth, try-in was done to evaluate both esthetics and occlusion (Figures 8(a) and 8(b)).

**Figure 9:**
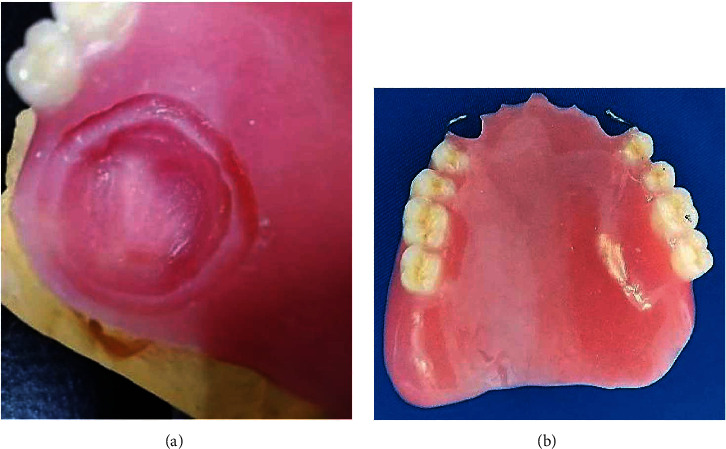
(a) Hollowing the prosthesis. (b) Prosthetic extrados aspect.

**Figure 10:**
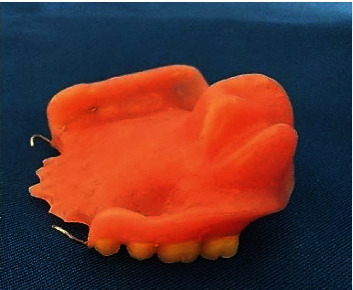
Obturator prosthesis.

**Figure 11:**
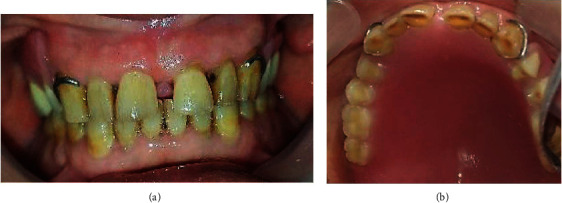
Mouth insertion: (a) frontal view; (b) occlusal view.

Once polymerized, the prosthesis was hollowed in the external surface, facing the obturator, to light it and prevent possible static instability (Figures 9(a) and 9(b)). Then, the prosthesis was polished (Figure 10) and inserted in mouth (Figures 11(a) and 11(b)).

The patient was advised to insert the prosthesis after repetitive trials in front of a mirror. Wearing and oral hygiene advice were instructed to the patient, and follow-up appointments were scheduled.

## 3. Discussion

Orofacial cancer therapeutics complicates prosthetic rehabilitation. Resection surgery modifies local anatomical conditions. All of these criteria, combined with complex psychological contexts, influence the prognosis of a successful prosthetic management [5].

The obturator prosthesis is a therapeutic option that is still used in many clinical cases where esthetic and functional rehabilitation cannot be achieved using another alternative [6]. It is an artificial device designed to ensure a tight closure of a bucconasal and/or buccosinusal communication. It is therefore considered as a complementary treatment to surgery and it requires some prerequisites [7].

The consequences of maxillary defects depend on their location and size [8]. In our case, the defect was of moderate size. The consequences faced with those cases are classified in Functional disorders, Infectious problems, Esthetic alterations, and Psychological and relational repercussions.

For the Functional disorders, we note speech difficulties due to nasal leakage of air, the voice is nasalized with incomprehensible words [8]; the alimentation becomes very disturbed by the reflux of food and liquids into the nasal and sinus fossae [9].

Infectious problems consist of chronic infection of the sinus cavities that may set in [9].

Regarding esthetic alterations, resection of the underlying bone leads to sagging of the soft tissue with deterioration of the patient's esthetics [9].

Concerning psychological and relational repercussions, since the maxillofacial complex is the seat of mimics of the organs of olfaction, vision, hearing and taste, and the origin of the respiratory and digestive tracts. This gives this anatomical part a major psychological and relational importance. Any mutilation to this part profoundly affects the patients and makes them vulnerable and isolated from their social environment [8, 9].

In order to avoid these severe and handicapping consequences, it is imperative to place an obturator prosthesis. This prosthesis can only be designed within a surgical-prosthetic symbiosis plan. Thus, before starting the treatment, a multidisciplinary consultation meeting involving the different specialists (maxillofacial surgeon, prosthodontist, radiologist, and social worker) must be held to outline the main lines of the treatment plan [4].

The definitive obturator prosthesis can be used for only 3 months to 1 year after the resection surgery. Dimensional changes due to remodeling and scarring of the defect's contours last approximately 1 year and are more related to soft tissue remodeling than to bone tissue remodeling. The decision for definitive rehabilitation depends on the size of the defect, the tumor prognosis, the mouth opening which must be sufficient, and the patient's edentulism. Healing is considered satisfying in the absence of sequestrum removal and in the presence of a well-reepithelialized and uninfected excision cavity [10].

In our case, a rigid obturator was indicated because the patient was partially edentulous and the maxillary defect was of moderate size (classification of M. Benoist). For the fabrication, the partial denture and the obturator were fabricated at the same time. Rigid obturator was made of methyl methacrylate. This material has the advantage of being durable in the long term, can be easily cleaned, and being perfectly polished. In addition, the weight of the denture can be reduced by hollowing the obturator [11]. For this, many materials can be used. For our case, it was the wax while salt or sand are also described for the same purpose in the literature. [12]

The impression of the maxillary defect was taken with FITT material (Kerr®) because it presented undercuts. It is a delayed-setting resin obtained by mixing powder and liquid and whose setting reaction goes through four phases: gel phase, plastic phase, elastic phase, and drying phase. Its plastic nature allowed us to mold the undercuts of the maxillary defect during the insertion of the custom tray. Its elasticity after setting allowed us to disinsert it without tearing or detachment of the impression [13].

Computer-assisted design and computer-assisted manufacturing (CAD/CAM) of obturators are also reported in the literature. Indeed, this method has many advantages, including saving time by reducing the number of steps and the possibility of avoiding some painful impressions by taking optical ones [14].

For many years, management in cancerology has been a controversial issue: should we surgically reconstruct following carcinological excision?

It is obvious that the surgical reconstruction rate is much higher. Nevertheless, we are still awaiting a scientific study on this subject to determine whether reconstruction compromises postsurgical monitoring and possibly modifies the survival time [15].

There are few comparative studies between the techniques of prosthetic rehabilitation and surgical reconstruction. Boutault et al. noted problems of food or fluid leakage in patients rehabilitated with obturators and phonatory problems in surgically reconstructed patients [16].

Based on a study involving 47 patients, Bertrand concluded that in the case of a maxillary defect with a size less than a quarter of the hard palate, the obturator prosthesis gives excellent functional results [17].

There is no doubt that prosthetic rehabilitation procedures still have an important role to play, especially in modest defects in the field of carcinology. On the other hand, there is a growing consensus that a severe defect is an indication for surgery. The choice of the best technique to be used remains rather subjective, as shown by numerous publications which are sometimes contradictory. It is sometimes possible to combine surgical and prosthetic approaches [15, 16].

Prosthetic materials can be improved and tissue engineering can be developed for the patients' benefit. There is no doubt that this subject is expected to evolve continuously and probably for quite a long time regarding its complexity [18].

## 4. Conclusion

Maxillofacial cancers most often require surgical excision of the developed tumors. This leads to a maxillary defect that can affect the esthetic balance of the face and have important consequences on the functional performances, especially, in case of a large bucconasal communication.

In spite of the evolution of surgical techniques, conventional obturator prostheses are still the gold standard to compensate maxillary defects.

## Data Availability

Data supporting this study are within the article.
